# Assessment of renal quality with quantitative contrast-enhanced ultrasound (CEUS) for differentiating kidney histopathology before procurement

**DOI:** 10.7150/ijms.88147

**Published:** 2024-01-01

**Authors:** Shangxin Dong, Bo Zhang, Zhishui Chen, Dunfeng Du, Huibo Shi, Yuanyuan Zhao, Yukun Tang, Hongchang Luo, Jipin Jiang

**Affiliations:** 1 Institute of Organ Transplantation, Tongji Hospital, Tongji Medical College, Huazhong University of Science and Technology; Key Laboratory of Organ Transplantation, Ministry of Education; NHC Key Laboratory of Organ Transplantation; Key Laboratory of Organ Transplantation, Chinese Academy of Medical Sciences, Wuhan, China.; 2 Department of Medical Ultrasound, Tongji Hospital, Tongji Medical College, Huazhong University of Science and Technology, Wuhan, China.

**Keywords:** contrast-enhanced ultrasonography, kidney transplantation, organ procurement, donor selection

## Abstract

**Purpose:** This study aimed to investigate the use of contrast-enhanced ultrasonography (CEUS) to assess the kidneys' quality before procurement.

**Methods:** This prospective study included 74 donors and 148 recipients of kidneys. 119 kidneys underwent quantitative analysis. Before organ procurement, potential kidney donors underwent CEUS, though organ procurement involved a zero-point puncture biopsy. CEUS parameters of the renal cortex and medulla were evaluated, including rise time (RT), time to peak (TTP), the area under the curve (AUC), wash-in slope (WIS), peak intensity (PI), and mean transit time (MTT). Donors' kidneys were classified based on their pathological. Additionally, short-term clinical indicators of renal recipients were collected and analyzed to determine whether the patients had delayed recovery of renal allograft function.

**Results:** This experiment included 148 cases of kidney information, divided into two groups based on the Remuzzi score of the kidneys. However, 29 kidneys were excluded from the quantitative analysis due to loss or low quality of CEUS images. Comparing the time-intensity curve (TIC) of renal cortical region of interest (ROI), we found that the group with lower pathological scores exhibited higher PI (P=0.002), AUC(P=0.003), and WIS (P=0.009). TIC comparison results for renal medulla ROI revealed that the group with lower pathological scores had higher PI (P=0.010), AUC (P=0.023), and WIS (P=0.024).

**Conclusions:** This study highlighted the potential of CEUS as a non-invasive, safe, and real-time examination method that correlates with the Remuzzi score and renal pathology. Therefore, it can be used as a prospective preoperative non-invasive evaluation method for the donor's kidney.

## Introduction

Kidney transplantation (KT) is regarded as one of the most efficient therapies for patients with end-stage renal failure. Since 2015, organ donation after death has been China's only lawful source of organs^1^. However, due to the acute organ shortage, expanded-criteria donors (ECD) have become a common method of expanding the donor pool^2^. The use of ECD has reduced the waiting time for kidney transplantation. Nevertheless, this method has increased the number of kidneys discarded, thereby wasting human and hospital resources. Although renal biopsy results play an important role in making acceptance decisions, it cannot predict the kidney's quality before procurement, resulting in unnecessary effort. Due to their invasive nature and prolonged cold ischemia time, pre-transplant biopsies increase the risk of potential organ loss, according to several studies^3^, ^4^. Additionally, renal biopsy results may also be affected by sampling bias^5^. Therefore, there is a need to discover a new method for evaluating renal quality in advance.

Color Doppler ultrasound is a convenient method to evaluate renal perfusion by measuring renal artery flow velocity and resistance index (RI) at the bedside. Contrast-enhanced ultrasonography (CEUS) is a viable alternative to color Doppler ultrasound for assessing the donor kidney's microcirculation perfusion when color Doppler ultrasound fails to do so. CEUS displays real-time perfusion employing microbubble contrast agents and complementary harmonic pulse sequences^6^. The microcirculation perfusion can be accurately reflected by analyzing the area under the curve of the region of interest. Furthermore, the ultrasound contrast agents lack nephrotoxicity and cannot be excreted by the kidneys, making them a safe and appropriate option for assessing renal microcirculation^7^. Currently, CEUS has been utilized in assessing various solid organs, including its application in identifying causes of graft dysfunction after kidney transplantation^8^-^10^. However, its effectiveness in evaluating the quality of donated kidneys remains uncertain. Before procurement, assessing the quality of marginal donor kidneys remains a formidable obstacle. This study aimed to examine the use of CEUS in evaluating the kidney quality of donors and predicting the results of the zero-point biopsy.

## Materials and Methods

### Ethics

A total of 148 donated kidneys obtained from October 2021 to October 2022 at Tongji Hospital were included in this study. These donor grafts were donated to the Red Cross Society of Hubei Province and allocated by the China Organ Transplant Response System. This investigation was conducted following China's national organ donation program and per the 2013 Helsinki Declaration^11^. All organ donors included in this study were adults. Exclusion criteria included allergies to contrast agents, hydronephrosis, renal artery stenosis, and malignant tumors. Patients who did not consent to participate were also excluded. The study was approved by the Ethics Committee of Tongji Hospital, affiliated with Tongji Medical College of Huazhong University of Science and Technology (TJ-IRB20220149). Informed consent was obtained from each recipient and each donor's immediate family members.

### US and CEUS examinations

Experienced radiologists who were skilled in urologic US and had more than 5 years of CEUS examination experience conducted all US/CEUS examinations on the donors. These doctors lacked access to the clinical and laboratory data of the patients, ensuring a blind study.

**Figure 1:**
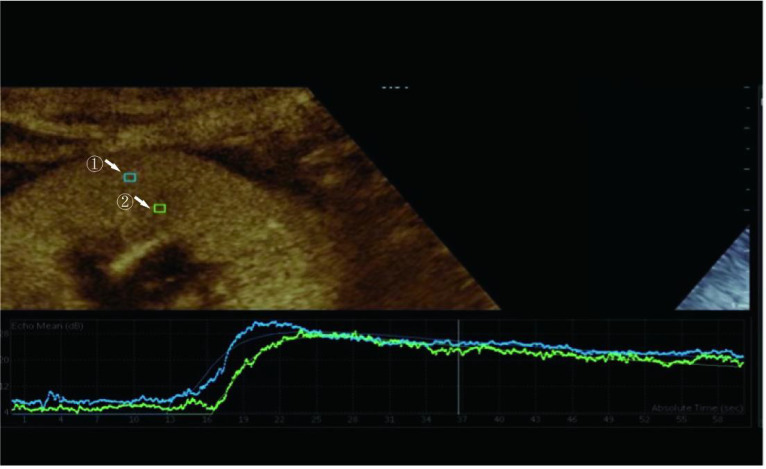
Region of interest selected for quantitative analysis. Region ① is the ROI of the renal cortex. Region ② is the ROI of the renal medulla.

We examined prospective donor kidneys at the bedside using CEUS within 24 h before procurement. The patient's blood pressure and the use of vasoactive drugs during the examination were noted. Philips EPIQ7 ultrasonic diagnostic instrument with a c5-1 probe was used for a contrast-enhanced ultrasound examination. The blood pool contrast agent used in this study was SonoVue (Bracco, Milan, Italy), containing sulfur-hexafluoride microbubbles stabilized by a phospholipid shell. Some studies have confirmed the safety of SonoVue^12^. First, both the left and right sides of the kidneys were scanned longitudinally and transversely with conventional US. The renal size, echo, and cortical thickness were recorded. After a typical US examination of the potential donors, we positioned the ultrasonic probe along the kidney's long axis to disclose the renal hilus and maximum kidney area. Then, 1.0 mL of SonoVue was injected via the central venous catheter, followed immediately by an infusion of 5 mL of the saline solution. Image acquisition began at the start of the SonoVue injection and recorded the acquired images on the local hard disk drive in the form of DICOM. During CEUS, the primary gain, focus position, time gain compensation, and other preset parameters remain unchanged. The mechanical index was set at 0.07 (Figure 1).

### Image analysis

The perfusion software Sonolive was used to conduct a quantitative analysis of the images. The 'motion compensation' function was enabled to reduce respiration's impact during the analysis. A 4 ^ 2 mm square region of interest (ROI) was placed in the cortex and medulla of the kidney, excluding the interlobar and arcuate arteries (Figure 1). An ROI's signal intensity was measured, and a time-intensity curve (TIC) was generated. Using the following parameters, two TICs were generated for each kidney: rise time (RT), time to peak (TTP), peak intensity (PI), wash-in slope (WIS), the area under the curve (AUC), and mean transit time (MTT). The quantitative ROI parameters of the cortex and medulla were analyzed further.

### Blood and Histopathological Examinations

Blood samples were collected on the day of the CEUS examination, and all clinical blood tests, including blood routine and renal function, were conducted in the clinical laboratory. After brain death or cardiopulmonary death, confirmed organ donors were determined. The donor's kidney was biopsied during the procurement process at the puncture site. The biopsied tissue is then quickly placed in a formaldehyde solution, embedded in paraffin, and sectioned for hematoxylin-eosin (HE) stains. The Remuzzi score^13^ and biopsy histological findings of the donor's kidney should be described. The study revealed several kidney histological findings, including interstitial fibrosis, tubular atrophy, renal interstitial inflammation, arteriolar intimal fibrosis and thickening, arterial hyaline degeneration, intraglomerular thrombosis, and acute renal tubular injury (Table 1).

### Statistical analysis

The data were examined using SPSS 26.0 (SPSS lnc., Chicago, USA) software, and demographic and clinical variables were presented as mean value ± standard deviations or median with range. The chi-square test was used to analyze differences between qualitative variables, whereas the one-tailed t-test or Wilcoxon test evaluated differences between quantitative variables. Univariate logistic analysis analyzed all these parameters. P<0.05 is considered statistical significance. By analyzing the ROC curve, the reliability of continuous measurement parameters was calculated. AUROC has been measured and the Youden test has been performed to find out the cut-off to maximize diagnostic accuracy.

## Results

### Demographics and Baseline Characteristics

Between October 2021 and October 2022, our institution conducted CEUS examinations on both kidneys before surgical procurement for 74 donors after brain death (DBD) or donation after cardiac death (DCD). Biopsies were performed on all donor's kidneys during procurement, yielding pathological information for 148 kidneys. The kidneys were then divided into two groups based on the Remuzzi score: the mild change group (≤ 3 points, n = 74) and the severe change group (≤ 4 points, n = 74). The demographic and clinical characteristics of these two groups are summarized in Table 2. Statistical analysis revealed significant differences in age, Cr, and eGFR between the two groups (Table 2, *p* < 0.05). The mild change group had higher eGFR and lower Cr levels than the severe change group. Moreover, the mild change group was (P < 0.05). However, there were no significant differences in gender and donor type between the two groups (P > 0.05). During the experiment, no adverse reactions were caused by the ultrasound contrast agent.

### The CEUS parameters showed significant differences between the different groups based on histopathological findings

The study utilized the Remuzzi score to classify the CEUS parameters into mild and severe change groups. Each kidney was analyzed for two TICs, including cortical and medullary TICs. However, 29 kidneys were excluded from the quantitative analysis due to image loss or poor quality. The mild change group comprised 61 cases, while the severe change group included 60 cases. The study found no significant differences in RTc, MTTc, and TTPc between both groups when comparing the CEUS parameters of renal cortex ROI (P > 0.05). However, there was a significant difference in PIc, AUCc, and WISc between the two groups (P < 0.05), as shown in Table 3. Additionally, when comparing the CEUS parameters of the renal medulla ROI, no significant difference was found in RTm, MTTm, and TTPm between the two groups (Table 3, P *>* 0.05). However, both groups significantly differed in PIm, AUCm, and WISm (Table 3, P < 0.05).

This study examined the relationship between CEUS parameters and renal pathology. We divided the parameters based on the specific pathological manifestation to analyze the data. Renal interstitial fibrosis and renal tubular atrophy were categorized into normal and mild groups. Similarly, arteriolar intimal fibrosis and arteriolar hyalinosis were divided into two groups. The first group included normal and mild cases, while the second included moderate and severe cases. Significant differences were found between the two groups based on renal interstitial fibrosis regarding AUCc and MTTm (P < 0.05). In the normal group, AUCc values were larger (680.46 VS 592.51 dB*s, P = 0.032), and MTTm was longer (27.11 VS 24.15 s, P = 0.048). Furthermore, renal tubular atrophy was found to be correlated with AUCc and MTTm, showing larger AUCc (660.67 VS 578.26 dB*s, P = 0.033) and longer MTTm (26.74 VS 23.17 s, P = 0.020) in the normal group. The CEUS parameters analysis based on the arteriolar intimal fibrosis grouping revealed that the mild change group had faster TTPm (21.23 s VS 24.70 s, P = 0.036). Comparing the parameters of CEUS based on the grouping of arteriolar hyalinosis, it was found that the group with mild changes had faster RTc (6.55 VS 7.70 s, P = 0.045), TTPc (15.82 s VS 19.44 s, P=0.011), and TTPm (19.90 VS 24.66 s, P = 0.004).

### CEUS parameters have significant correlation with Remuzzi score

This study utilized logistic regression analysis to determine which parameters of CEUS accurately predicted the severity of pathological conditions. Table 5 demonstrates that according to univariate logistic regression analysis, PIc, AUCc, WISc, PIm, AUCm, and WISm were all significantly associated with the severity of renal pathology (P < 0.05).

**Figure 2:**
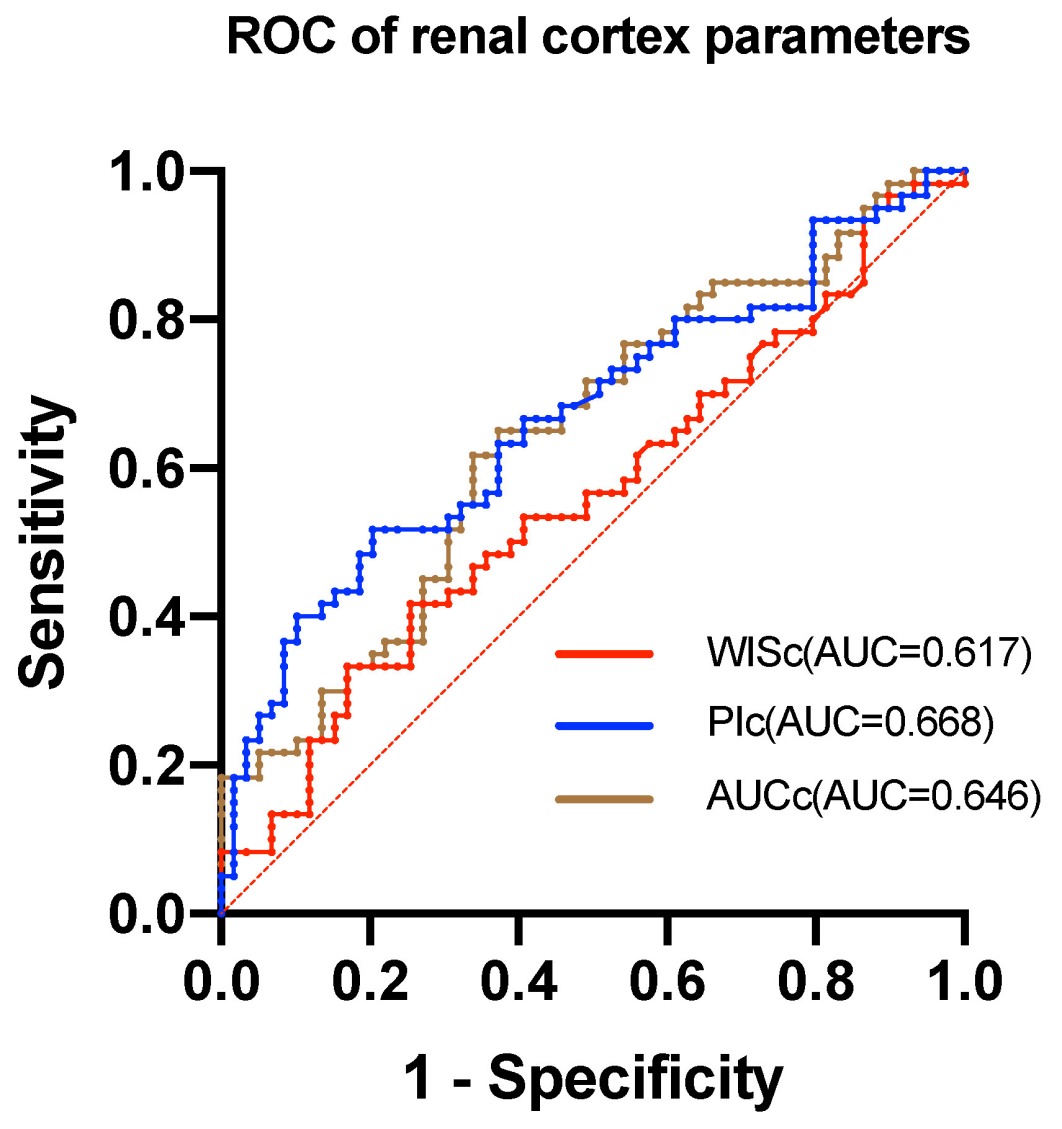
The ROC of renal cortex parameters. The AUC of WISc is 0.617. The AUC of PIc is 0.668. The AUC of AUCc is 0.646.

**Figure 3:**
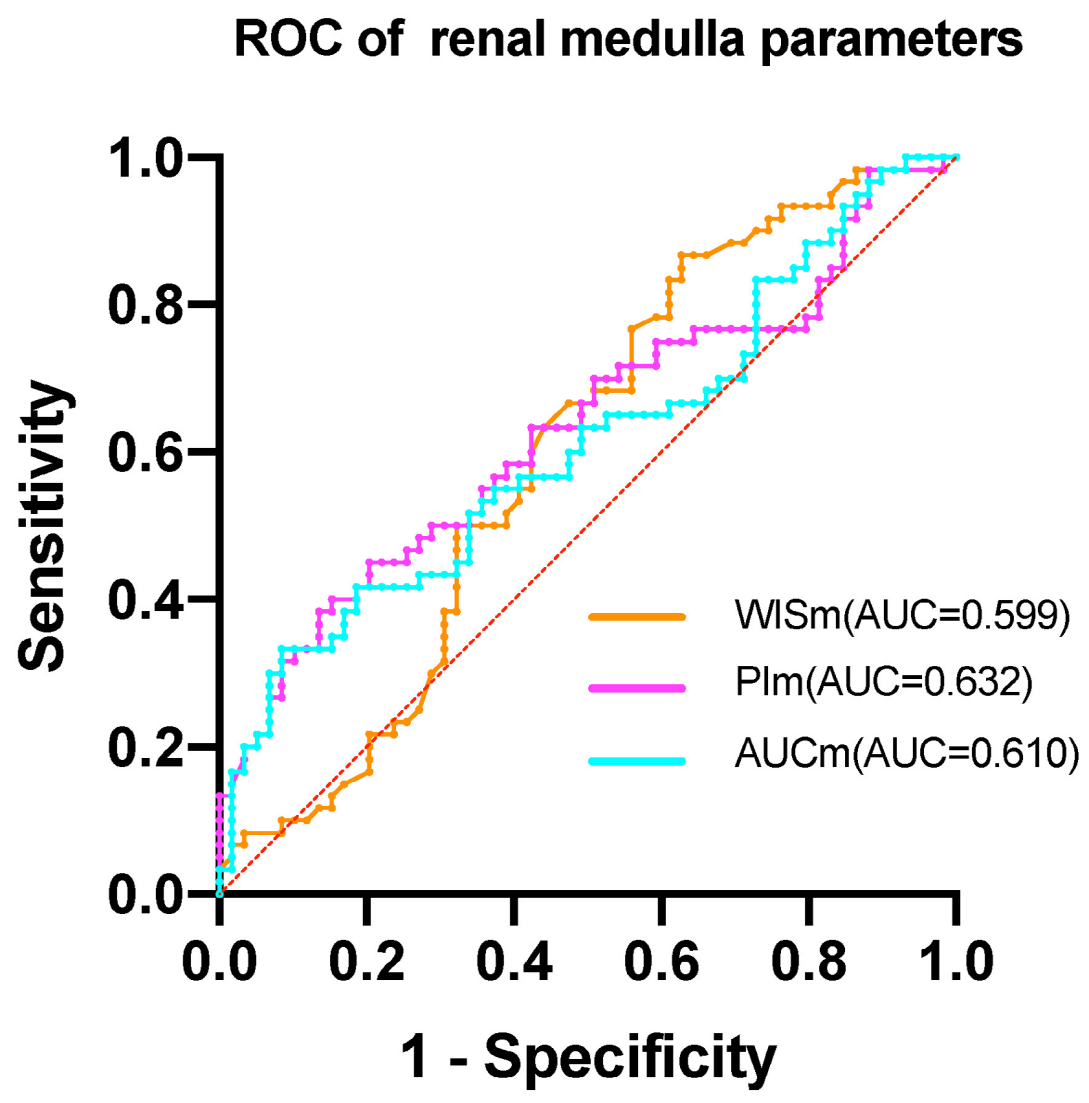
The ROC of renal medulla parameters. The AUC of WISm is 0.599. The AUC of PIm is 0.632. The AUC of AUCm is 0.610.

The study found that the AUROC of WISc was 0.617 (Table 6), while the PIc AUROC was 0.668 (Figure 2). The AUROC for the area under the TIC of the renal cortex ROI was 0.646, and the PIm AUROC was 0.632 (Figure 3). Additionally, the AUROC for the area under the TIC of the renal medulla ROI was 0.610. The PIc cutoff (Youden index) of 14.75 dB was determined based on the highest sensitivity (51.7%) and specificity (79.7%) for discriminating between the mild and severe change groups.

### CEUS parameters have no significant difference between delayed graft function (DGF) group and normal group

To examine the correlation between CEUS parameters and the recovery of renal allograft function after surgery, we gathered postoperative clinical data from patients at our institution. Fourteen kidneys involved in dual or combined organ transplants were excluded. However, the DGF group comprised the patients whose serum creatinine value did not drop below 400umol/L in the first postoperative week and required hemodialysis intervention^14^. Patients with normal renal allograft function comprised the normal group, and CEUS parameters were compared between the normal and abnormal renal allograft function groups. As shown in Table 7, no statistically significant differences were observed between the two groups.

## Discussion

The most widely used method for evaluating the quality of kidney grafts is the Kidney Donor Profile Index (KDPI). Studies have shown a negative correlation between KDPI and graft survival rate^15^, ^16^. The relationship between procurement biopsy results and transplantation results is still uncertain. A Systematic review on the prediction of transplantation results from donor kidney biopsy results found that there was no consistent relationship between donor biopsy results and transplantation results^17^. Nevertheless, purchasing biopsy results still plays an important role in determining whether the kidney is accepted^18^. The histological information of the donor kidney is often used to determine the rejection, allocation, and whether to undergo dual kidney transplantation.

CEUS facilitates objective quantitative and comparative analysis of organ tissue perfusion. Using CEUS parameters and pathological data from the donor's kidneys, significant differences in PIc, AUCc, WISc, PIm, AUCm, and WISm were observed between pathological groups. Additionally, univariate logistic regression analysis revealed that AUCc, TTPc, MTTm, and TTPm were significantly associated with renal pathology severity (P < 0.05). The mean values of PI, AUC, and WIS for the Remuzzi score were greater in the mild change group than in the severe change group. Also investigated was the correlation between CEUS parameters and short-term graft function recovery in renal transplant recipients. Following the operation, patient clinical data were collected, and renal transplant patients were categorized as DGF and normal groups. Our study found no significant differences between the two groups in terms of CEUS parameters. However, a strong correlation between CEUS, Remuzzi scores, and renal pathology has been observed. Thus, it indicates that CEUS has the potential to be used as a non-invasive method for evaluating donor kidney preoperative assessment.

ROC curves were generated to evaluate the diagnostic value of ultrasound contrast parameters associated with the Remuzzi score. The diagnostic threshold for the optimal indicator PIc was calculated and found to be 14.75 dB, indicating that PIc values below this cutoff could benefit diagnosis. In the mild and severe change groups, a sensitivity of 51.7% and a specificity of 79.2% were observed. Although the current diagnostic efficacy of CEUS parameters cannot fully replace histopathology examination, it remains a promising non-invasive technology for assessing donor kidneys' quality before procurement.

Quantitative CEUS has been investigated to distinguish histopathological damage following renal transplantation. In Eva Vi's study, transplanted kidneys were categorized according to the Banff classification, including the minimal change and significant injury groups^19^. After analyzing the CEUS parameters of the two groups, it was determined that the difference in time to peak between the medulla and cortex (TTP m-c) is an effective diagnostic tool for predicting severe pathological damage to transplanted kidneys. In a recent investigation on donor livers, 67 were examined using CEUS before surgical procurement. Of these,15 livers (22.4%) were discarded, while 52 (77.6%) were deemed transplantable^20^. According to multivariate analysis, a decrease in enhancement (OR = 2.588, 95% CI: 1.234-5.426, P = 0.012) was an independent factor for liver discard. Furthermore, a study that utilized quantitative parameters of CEUS to evaluate the pathology of chronic kidney disease found that the average values of PI and AUC decreased as the severity of the disease increased^21^. The study found no correlation between RT and WIS with kidney pathology (P > 0.05). However, another investigation revealed that CEUS parameters such as peak enhancement, wash-in and wash-out AUC were substantially different between patients with diabetes nephropathy (DN) and those with non-diabetes renal disease (NDRD). It was identified during a CEUS evaluation of renal microcirculation perfusion in patients with renal injury and diabetes^22^. Peak enhancement and AUC may be potential parameters to differentiate DN.

Therefore, further research is required to provide additional evidence. CEUS is generally a promising screening tool for marginal donor kidneys. Predicting kidney quality using CEUS is critically essential for organ acquisition. Currently, renal puncture biopsy is the most accurate technique for assessing kidney health^23^. However, the cost of rejecting kidneys due to substandard quality after acquisition raises the manpower and material costs associated with organ procurement. Prior knowledge of kidney pathology and preoperative evaluation can improve resource allocation and decrease acquisition costs.

This research has limitations that must be acknowledged. First, although experienced ultrasound physicians performed CEUS, the consistency between observers was not evaluated by two independent technicians. Second, no kidneys were discarded in this study due to inadequate quality, and no donor's kidneys were discarded for comparison. Third, during TIC analysis, the patient's respiration can significantly interfere with the parameters, particularly when the ROI receives signals from small arteries. Additionally, the use of vasoactive drugs in the ICU before surgery by most potential dead donors can affect the results of CEUS.

In conclusion, this study found a correlation between renal PI, AUC, and WIS and renal pathology. CEUS is indicated as a potential technique for evaluating renal pathology prior to surgery.

## Figures and Tables

**Table 1:** Standard used to evaluate renal pathology.

Pathological feature	Normal	Mild	Moderate		severe
Interstitial fibrosis	<5%	5-25%		26-50%	>50%
Tubular atrophy	<5%	5-25%		26-50%	>50%
Interstitial inflammation	<5%	5-25%		26-50%	>50%
Arteriolar intimal fibrosis	<5%	5-25%		26-50%	>50%
Arteriolar hyalinosis	No found	At least one small artery is involved		Multiple small arteries are involved	Hyalinosis totally around multiple small arteries
Glomerular thrombi	No found	<10%		10-25%	>25%
Acute tubular necrosis	No found	Swelling of renal tubular epithelium, nuclear shedding, brush border disappearance		Focal coagulative necrosis of renal tubular epithelium	Massive ischemic necrosis
					

**Table 2:** Donor data of mild change and non-mild change groups

Parameter	Mild change group (n=74)	Severe change group (n=74)		*P*
Sex				
Male	63	67	0.314	
Female	11	7		
Age (years)	51.1±12.6	56.5±11.0	0.006	
Weight (Kg)	67.6±16.2	67.6±14.4	0.987	
BMI (Kg/m)	23.1±4.9	23.0±4.1	0.944	
Type				
DBD	20	22	0.715	
DCD	54	52		
SCr (μmol/L)	74.0 (58.0-95)	87.0 (68.7-125)	0.002	
eGFR (mL/min/1.73 m2)	96.7±28.3	77.3±30.0	<0.001	
Hb (g/L)	99.8±21.5	103.6±26.1	0.487	
Conventional US data				
Renal length (mm)	10.85±1.41	10.58±0.86	1.000	
Cortical thickness(mm)	0.91±0.47	0.83±0.15	0.143	
				

BMI: body mass index; DBD: donation after brain death; DCD: donation after cardiac death; SCr: serum creatinine; eGFR: estimate glomerular rate; Hb: hemoglobin.

**Table 3:** Comparison of CEUS parameters of kidney between mild change group and non-mild change group

Parameter	Mild change group(n=59)	Severe change group(n=60)	P
RTc	7.39±3.48	7.15±2.62	0.670
PIc	18.39±4.45	15.73±4.59	***0.002***
AUCc	689.77±229.97	571.62±198.33	***0.003***
WISc	2.42±1.08	1.96±0.81	***0.009***
MTTc	23.78±6.16	23.05±5.57	0.497
TTPc	16.94±6.95	19.18±8.79	0.126
RTm	9.99±4.90	8.56±3.39	0.067
PIm	14.91±4.73	12.65±4.70	***0.010***
AUCm	563.02±237.13	468.38±209.69	***0.023***
WISm	1.51±0.79	1.22±0.53	***0.024***
MTTm	26.27±9.42	24.58±6.50	0.255
TTPm	22.00±8.68	23.71±9.14	0.299

**RTc** rise time of TIC of kidney cortex, **PIC** peak intensity of TIC of kidney cortex, **AUCc** area under the TIC of kidney cortex, **WISc** wash in slope of TIC of kidney cortex, **MTTc** mean transit time of kidney cortex, **TTPc** time to peak of TIC of kidney cortex, **RTc** rise time of TIC of kidney medulla, **PIc** peak intensity of TIC of kidney medulla,** AUCc** area under the TIC of kidney medulla, **WISc** wash in slope of TIC of kidney medulla, **MTTc** mean transit time of kidney medulla, **TTPc** time to peak of TIC of kidney medulla.

**Table 4:** Comparison of CEUS parameters according to difference pathological feature.

Parameter	Interstitial fibrosis	Tubular atrophy	Arteriolar intimal fibrosis	Arteriolar hyalinosis
RTc	P=0.905	P=0.644	P=0.995	***P=0.045***
PIc	P=0.110	P=0.050	P=0.110	P=0.741
AUCc	***P=0.032***	***P=0.033***	P=0.086	P=0.634
WISc	P=0.437	P=0.686	P=0.055	P=0.071
MTTc	P=0.126	P=0.201	P=0.496	P=0.260
TTPc	P=0.515	P=0.222	P=0.129	***P=0.011***
RTm	P=0.242	P=0.067	P=0.389	P=0.887
PIm	P=0.136	P=0.148	P=0.115	P=0.350
AUCm	P=0.086	P=0.115	P=0.109	P=0.188
WISm	P=0.567	P=0.686	P=0.130	P=0.156
MTTm	***P=0.048***	***P=0.020***	P=0.702	P=0.803
TTPm	P=0.716	P=0.222	***P=0.036***	***P=0.004***

**Table 5:** Logistic regression analysis of CEUS parameters

Parameter	OR (95%)	*P*
RTc (s)	0.974 (0.866, 1.097)	0.668
PIc (dB)	0.876 (0.803, 0.956)	***0.003***
AUCc (dB s)	0.997 (0.995, 0.999)	***0.005***
WISc (dB/s)	0.595 (0.397, 0.892)	***0.012***
MTTc (s)	0.979 (0.920, 1.041)	0.494
TTPc (s)	1.037 (0.990, 1.087)	0.128
RTm (s)	0.920 (0.841, 1.007)	0.070
PIm (dB)	0.900 (0.828, 0.979)	***0.014***
AUCm (dB s)	0.998 (0.996, 1.000)	***0.027***
WISm (dB/s)	0.512 (0.293, 0.928)	***0.027***
MTTm (s)	0.974 (0.931, 1.019)	0.253
TTPm (s)	1.022 (0.981, 1.065)	0.297

**Table 6:** ROC analysis to evaluate the discrimination ability of CEUS parameters in renal pathology.

Parameter	AUROC	95%CI	P value
WISc	0.617	0.516-0.717	0.028
PIc	0.668	0.571-0.766	0.002
AUCc	0.646	0.548-0.745	0.006
WISm	0.599	0.495-0.702	0.063
PIm	0.632	0.531-0.732	0.013
AUCm	0.610	0.509-0.712	0.038

**Table 7:** Comparison of CEUS parameters of kidney between DGF group and NDGF group

Parameter	DGF group (n=22)	NDGF group (n=83)	*P*
RTc (s)	7.81±4.46	7.16±2.67	0.384
PIc (dB)	17.15±4.36	17.28±4.77	0.912
AUCc (dB s)	626.81±202.47	644.82±230.56	0.739
WISc (dB/s)	2.35±1.12	2.18±0.94	0.480
MTTc (s)	23.64±5.35	23.65±6.13	0.998
TTPc (s)	18.32±8.68	17.78±7.67	0.777
RTm(s)	9.63±5.34	9.02±3.82	0.545
PIm(dB)	13.34±4.48	14.20±4.87	0.454
AUCm (dB s)	492.50±200.87	538.73±236.11	0.403
WISm(dB/s)	1.39±0.91	1.39±0.62	0.997
MTTm(s)	25.93±8.15	25.42±8.08	0.795
TTPm(s)	25.12±9.66	21.92±8.09	0.117
